# Radioprotection in interventional cardiology: a step-by-step ‘call to action’ to promote gender equity

**DOI:** 10.1093/ehjimp/qyaf074

**Published:** 2025-06-09

**Authors:** Chiara Bernelli, Stefania Angela Di Fusco, Roxana Mehran, Furio Colivicchi

**Affiliations:** Cardiology Unit, Santa Corona Hospital, ASL2 Liguria, Via XXV Aprile 38, Pietra Ligure, SV 17027, Italy; Clinical and Rehabilitation Cardiology Unit, Emergency Department, San Filippo Neri Hospital, ASL Rome 1, Rome, Italy; Center of Interventional Cardiovascular Research and Clinical Trials ICAHN School of Medicine at Mount Sinai The Zena and Michael A. Wiener Cardiovascular Institute One Gustave L. Levy Place, PO Box 1030, New York, NY 10029-6574, USA; Clinical and Rehabilitation Cardiology Unit, Emergency Department, San Filippo Neri Hospital, ASL Rome 1, Rome, Italy

Despite widespread recognition of the occupational health hazards of^1,2^ radio-exposed workers, no concurrent proportional investment in research on radio-exposure risks and radioprotection measures has been implemented in interventional cardiology laboratories.

Furthermore, gaps exist regarding gender-specific radiation-induced diseases.^2^ Knowledge in this field can help reduce gender disparities in interventional cardiology.

To collect information on the perception of occupational radio-exposure hazards, the clinical research network of the ANMCO NEXT generation project^3^ devised a survey addressed to Italian professionals, both physicians and non-physicians, working in interventional cardiology facilities.^4,5^ The study showed that females (48% of the 237 workers who responded to the survey) more often than males, regardless of professional role (physicians or non-physicians), perceive gender discrimination in career progression (77.2% of females vs. 30.9% of males, *P* < 0.001) and work remuneration (49.1% vs. 17.1%, *P* < 0.001). Furthermore, females more than males expressed the need for more education on radioprotection (78.0% vs. 62.6%, *P* 0.009). In addition, allied healthcare workers, compared with medical staff, report a greater knowledge of laws regulating access to laboratory during pregnancy (93.5% vs. 48.3%, *P* < 0.0001). Overall, the survey results show that females and males differently perceive gender-based disparity in career opportunities. These results are consistent with previous studies in this field^2^ and warrant initiatives aimed at increasing knowledge on radioprotection and reducing the unfavourable impact of working radio-exposed on personal/family and professional life.^4-8^

The potential increased risk of cancer in more radiosensitive areas, such as breast, ovarian, prostate, thyroid, haematopoietic system, and brain is a further point that needs to be better defined in radio-exposed workers. Indeed, occupational radiation exposure has been recognized as a risk factor for breast cancer^9^ and brain neoplasms of the left side.^10^ These latter are related to the fact that, in interventional cardiologists, the left side of the head is generally more exposed to radiation than the right side.

Based on these premises a ‘call to action’ to expand knowledge, educate, and better protect radio-exposed healthcare professionals must be launched (*Figure 1*). The medical staff of different specialties, healthcare institutions, and scientific societies should collaborate to create solid epidemiological data on the hazards of radio-exposed workers and better define the impact on cancer risk, atherosclerotic cardiovascular disease risk, and fertility. Overall, the findings of these studies (*Figure 2*) would support the development of refined preventive strategies to contain radiological risk and follow-up programs to identify specific radio-induced pathologies early on.

**Figure 1:**
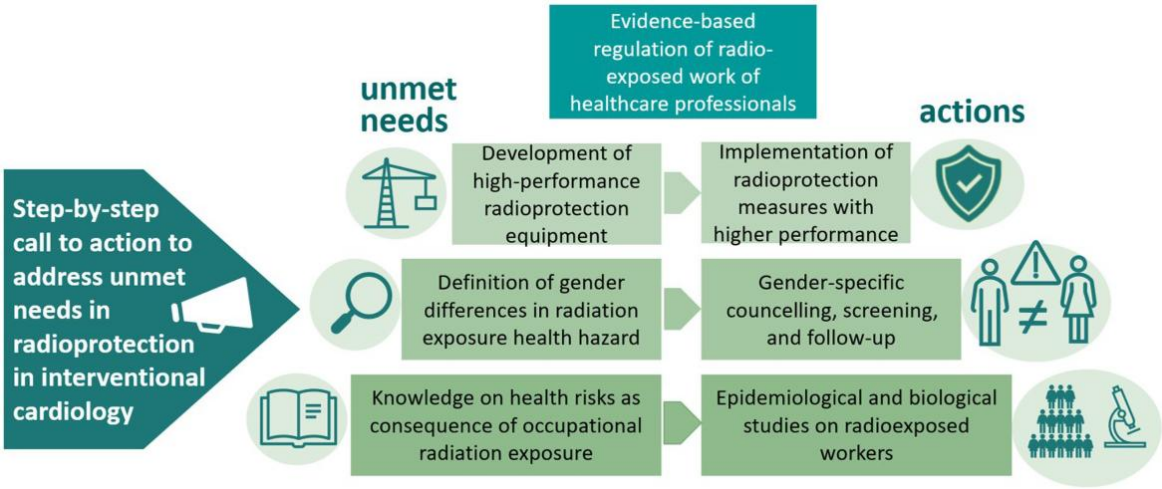
Needs and solutions to reduce the health hazard due to occupational radiation exposure.

**Figure 2:**
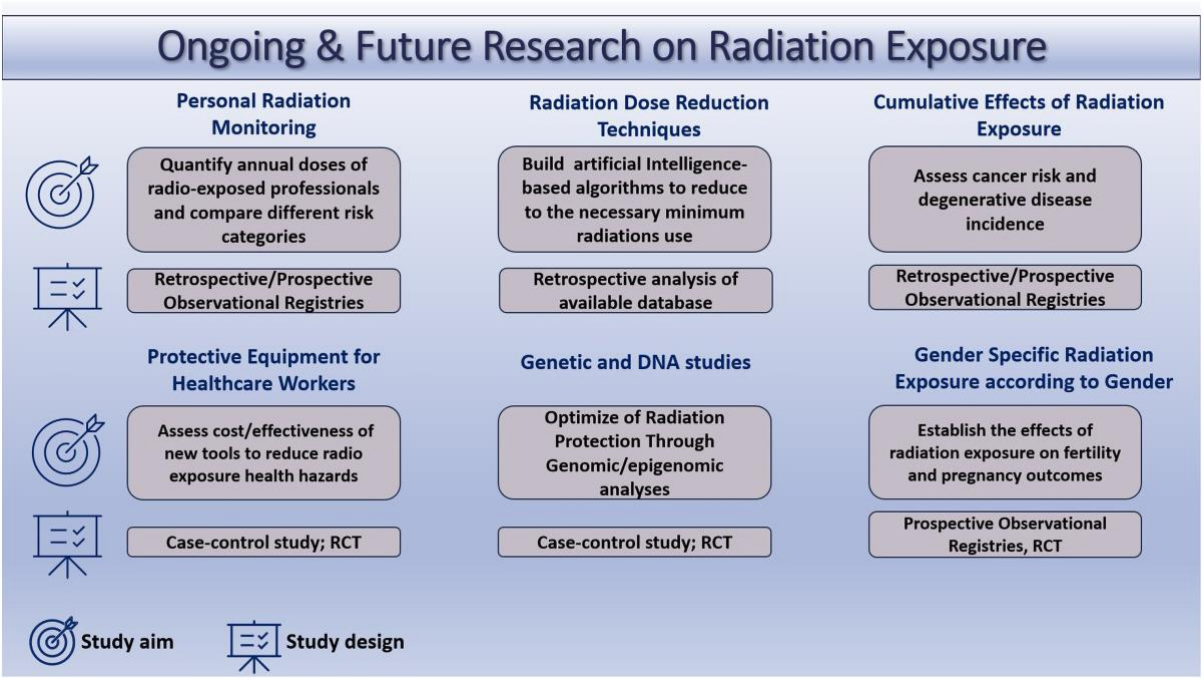
Area deserving further research and possible study design. RCT stands for randomized clinical trial.

To overcome potential gender disparities in career opportunities, particularly for pregnant cardiovascular trainees, as recently highlighted by Haghighat *et al*.^11^ the research should be focused on defining sex-specific risks and differences in radiation exposure.^12^ The potential risk of infertility related to working radio-exposed, as well as the potential fetus risks in cases of radio-exposed pregnancy, is not yet completely established. For example, a small increase in malformation and cancer risk in the offspring has been reported as a potential long-term effect of radiation exposure during pregnancy.^8^ Even though more evidence in these fields is warranted, national and international regulatory authorities have established specific rules for radio-exposed work during pregnancy. EU directives indicate 1 mSv as a safe limit dose of workers’ radiation exposure during pregnancy. However, currently, some European countries apply these directives in a restrictive manner and forbid working radio-exposed during pregnancy. These laws are further possible reasons that discourage female access to interventional cardiology subspecialties.^8^ Conversely, in the USA, where pregnant females can work in interventional cardiology, the limit dose of radiation during gestation is 5 mSv. Except for pregnancy-related risks, the safety standards for occupational radiation exposure currently do not consider potential gender-related health risk differences and represent additional issues to address. Furthermore, radioprotection counselling, screening, and follow-up should also consider gender-specific needs (*Figure 3*).

**Figure 3:**
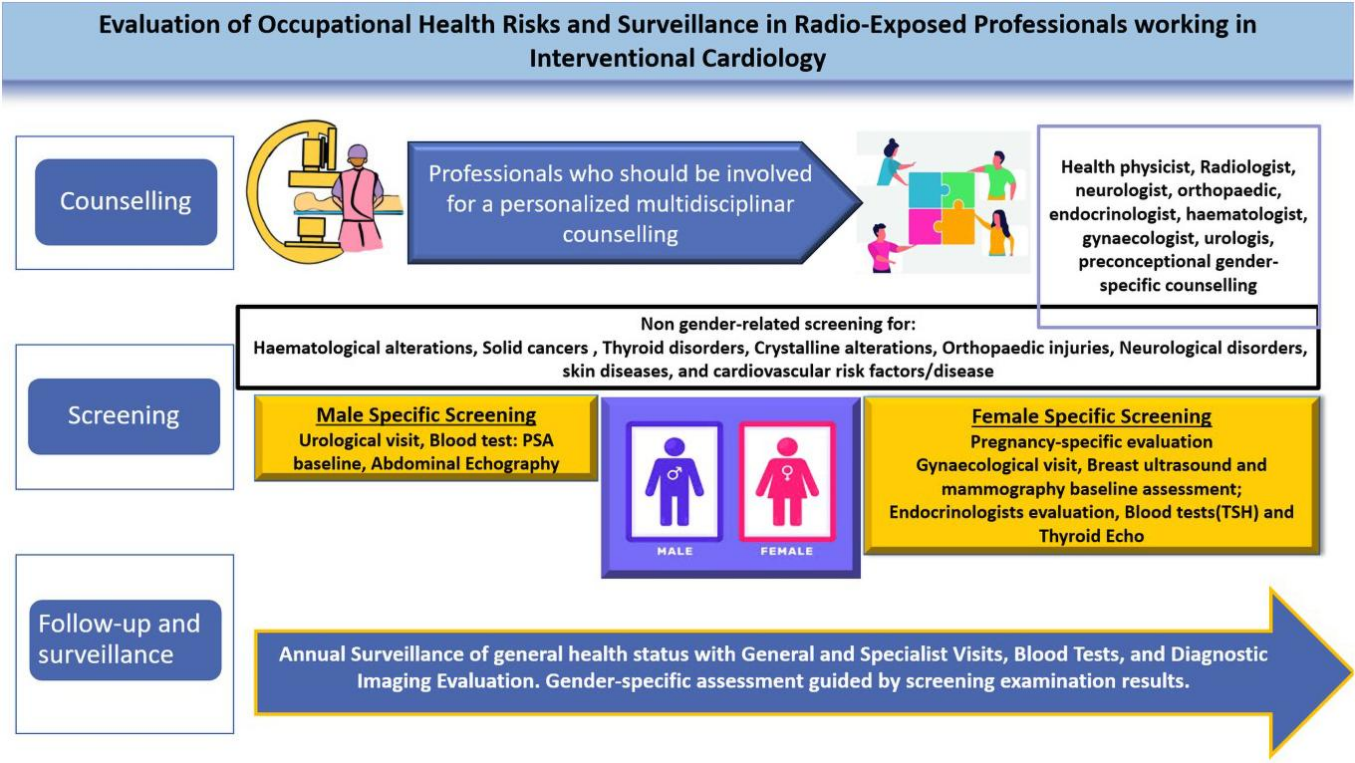
Counselling, screening, and follow-up in occupational radio-exposed healthcare professionals in interventional cardiology laboratories with a focus on gender.

Finally, technological research should deal with the development of new tools to reduce the radiation dose and to increase the protection from radiations to which operators and patients are exposed. Although some steps ahead in this direction have been made, currently the applicability and diffusion of such tools remain low due to the high costs. Therefore, in this setting, the next step is to raise the awareness of public decision-makers on the health impact of these tools and promote investment in this equipment to more effectively protect radio-exposed subjects.

Overall, in a cardiology era characterized by a growing diffusion of ionized radiation-guided procedures, a greater awareness of radiation exposure impact on healthcare professionals and the promotion of research on radioprotection represent key objectives to pursue. Working in safer interventional cardiology laboratories that protect females as well as males will help to reduce the gender gap.

## Lead author biography

Chiara Bernelli is a Cardiologist Specialist in Interventional Cardiology at ASL2, Santa Corona Hospital in Pietra Ligure (SV). Graduated in Medicine and Surgery and specialized in Cardiology at the University of Genoa with top marks (110 cum laude and Medal and 50/50), she completed her training experience by obtaining a Master's Degree in Interventional Cardiology II level at the San Raffaele Hospital in Milan. She worked at the San Raffaele Hospital in Milan, Papa Giovanni XXIII Hospital in Bergamo and at the ASST Nord Milano as a Cardiologist and Interventional Cardiologist. She is particularly interested in clinical research, collaborates in international and Italian clinical studies, as well as being the author of several publications and reviewer for international journals. She actively participates as a faculty in numerous national and international scientific conferences. She is an active member of the Italian Interventional Society (GISE) where she plays an active role in the Scientific Editorial Committee. She is deputy editor of the journal Minerva Cardioangiologica. She is a SICI-GISE fellow, has played an active role in the Italian board (GISE Young), as well as a member of the national association of hospital cardiologists ANMCO. She is also a member of the following international societies ESC, EAPCI WIN. One of the main research topics concerns interventional cardiology and gender with implications related to the gender gap and radiation protection.

## References

[1] Picano E, Santoro G, Vano E. Sustainability in the cardiac Cath lab. Int J Cardiovasc Imaging 2007;23:143–7. 17033729 10.1007/s10554-006-9148-x

[2] Bernelli C, Cerrato E, Ortega R, Piccaluga E, Ricottini E, Chieffo A et al Gender issues in Italian catheterization laboratories: the gender-CATH study. J Am Heart Assoc 2021;10:e017537. 33618540 10.1161/JAHA.120.017537 PMC8174252

[3] Di Fusco SA, Maggioni AP, Colivicchi F. A training project to promote clinical research among young cardiologists. Eur Heart J 2022;43:2005–7. 35246680 10.1093/eurheartj/ehac074

[4] Bernelli C, Di Fusco SA, Scicchitano P, Gatto MC. Radioesposizione e questioni di genere nei laboratori di cardiologia interventistica italiani. G Ital Cardiol 2023;24:1014–5. 10.1714/4139.41350 38009356

[5] Bernelli C, Di Fusco SA, Matteucci A, Zilio F, Nesti M, Barbero U et al Working in interventional cardiology laboratories: the perceived impact of radiation exposure as a health and gender hazard. A NEXT generation ANMCO initiative. Int J Cardiol 2024;401:131682. 38176657 10.1016/j.ijcard.2023.131682

[6] Gualano SK, Henderson J, Menees S, Kerkar A, Parisi E, Kerr EA. Women's representation in interventional cardiology. JAMA Cardiol 2024;9(9): 859–61. 38985493 10.1001/jamacardio.2024.1724 PMC11238065

[7] Abdulsalam N, Gillis AM, Rzeszut AK, Yong CM, Duvernoy CS, Langan MN et al Gender differences in the pursuit of cardiac electrophysiology training in North America. J Am Coll Cardiol 2021;78:898–909. 34446162 10.1016/j.jacc.2021.06.033

[8] Saada M, Sanchez-Jimenez E, Roguin A. Risk of ionizing radiation in pregnancy: just a myth or a real concern? Europace 2023;25:270–6. 36125209 10.1093/europace/euac158 PMC10103573

[9] Pilkington I, Sevenoaks H, James E, Eastwood D. Protecting female healthworkers from ionising radiation at work. BMJ 2023;381:e075406. 37045449 10.1136/bmj-2023-075406

[10] Roguin A, Goldstein J, Bar O. Brain tumours among interventional cardiologists: a cause for alarm? Report of four new cases from two cities and a review of the literature. EuroIntervention 2012;7:1081–6. 22207231 10.4244/EIJV7I9A172

[11] Haghighat L, Kalantarian S, DesJardin JT, Pellegrini CN. Evaluating pregnancy safety during cardiology training. JAMA Cardiol 2024;9:946–7. Erratum in: JAMA Cardiol. 2024 Sep 11. doi: 10.1001/jamacardio.2024.3111. 39110472 10.1001/jamacardio.2024.2294 PMC11307157

[12] Committee to Assess Health Risks from Exposure to Low Levels of Ionizing Radiation; Nuclear and Radiation Studies Board, Division on Earth and Life Studies, National Research Council of the National Academies. Health Risks from Exposure to low Levels of Ionizing Radiation: BEIR VII Phase 2. Washington, DC: The National Academies Press; 2006. 25077203

